# Endocannabinoids and related lipids in blood plasma following touch massage: a randomised, crossover study

**DOI:** 10.1186/s13104-015-1450-z

**Published:** 2015-09-29

**Authors:** Lenita Lindgren, Sandra Gouveia-Figueira, Malin L. Nording, Christopher J. Fowler

**Affiliations:** Department of Integrative Medical Biology, Umeå University, Umeå, Sweden; Pharmacology Unit, Department of Pharmacology and Clinical Neuroscience, Umeå University, Umeå, Sweden; Department of Chemistry, Umeå University, Umeå, Sweden

**Keywords:** Endocannabinoid, Palmitoylethanolamide, Oleoylethanolamide, Linoleoyl ethanolamide, Touch massage, Anxiety, Stress, Blood plasma, Perceived pleasantness

## Abstract

**Background:**

The endocannabinoid system is involved in the regulation of stress and anxiety. In a recent study, it was reported that short-term changes in mood produced by a pleasant ambience were correlated with changes in the levels of plasma endocannabinoids and related *N*-acylethanolamines (Schrieks et al. PLoS One 10: e0126421, 2015). In the present study, we investigated whether stress reduction by touch massage (TM) affects blood plasma levels of endocannabinoids and related *N*-acylethanolamines.

**Results:**

A randomized two-session crossover design for 20 healthy participants was utilised, with one condition that consisted of TM and a rest condition as control. TM increased the perceived pleasantness rating of the participants, and both TM and rest reduced the basal anxiety level as assessed by the State scale of the STAI-Y inventory. However, there were no significant effects of either time (pre- vs. post-treatment measures) as main effect or the interaction time x treatment for the plasma levels of the endocannabinoids anandamide and 2-arachidonoylglycerol or for eight other related lipids. Four lipids showed acceptable relative reliabilities, and for two of these (linoleoyl ethanolamide and palmitoleoyl ethanolamide) a significant correlation was seen between the TM-related change in levels, calculated as (post-TM value minus pre-TM value) − (post-rest value minus pre-rest value), and the corresponding TM-related change in perceived pleasantness.

**Conclusions:**

It is concluded that in the participants studied here, there are no overt effects of TM upon plasma endocannabinoid levels. Possible associations of related *N*-acylethanolamines with the perceived pleasantness should be investigated further.

**Electronic supplementary material:**

The online version of this article (doi:10.1186/s13104-015-1450-z) contains supplementary material, which is available to authorized users.

## Background

For centuries, extracts of the *Cannabis sativa* plant have been used for their therapeutic and recreational properties. ∆^9^-Tetrahydrocannabinol, the main psychoactive ingredient of cannabis produces its effects via activation of two cannabinoid (CB) receptors, CB_1_ and CB_2_. CB_1_ receptors are the most abundant G protein-coupled receptor expressed in the brain but are also found in cells and tissues outside the central nervous system. CB_2_ receptors are mainly expressed in cells of the immune tissue but have also been identified in the CNS [1, 2]. The most well-characterised endogenous CB receptor ligands (endocannabinoids, eCBs) are the arachidonic acid derivatives anandamide (AEA) [3] and 2-arachidonglycerol (2-AG) [4, 5]. AEA belongs to the *N*-acylethanolamine (NAE) class of lipids, which includes other putative eCB ligands such as docosatetraenoyl ethanolamide (DEA) [6] as well as the anti-inflammatory compounds palmitoylethanolamide (PEA) and stearoylethanolamide (SEA) [7, 8], the satiety factor oleoylethanolamide (OEA) [9] and other less well-characterised compounds such as linoleoyl ethanolamide (LEA), palmitoleoyl ethanolamide (POEA) and eicosapentaenoyl ethanolamide (EPEA). NAEs are catabolised by hydrolysis to the corresponding long-chain fatty acids, but AEA can also be metabolised to other compounds including *N*-arachidonoylglycine (NA-Gly) [10], which has biological properties of its own [11].

The endocannabinoid (eCB) system is involved in a wide variety of effects in the body, including the inhibition of central transmitter release and the regulation of appetite and pain perception [12]. A substantial amount of research has linked the eCB system to stress, fear and anxiety. One hypothesised function for the eCB system is to buffer or dampen the endocrine and behavioural effects of acute stress and negative stimuli [13, 14] and in humans increased levels of eCB have been detected in serum after exposure to acute stress [15]. Both decreases [16] and increases [17] in plasma eCB levels have been reported for individuals with post-traumatic stress disorder. Loss of eCB tone can result in mood disturbances in man: the CB_1_ receptor antagonist/inverse agonist rimonabant was briefly marketed for weight reduction. The medication was effective, patients lost weight and experienced food less pleasurably. However, after a short time on the market, the drug was withdrawn due an unfavourable risk of developing depressive disorders, mood alterations with depressive symptoms, and anxiety [18].

In a recent study [19], it was reported that in healthy women, the eCB system was responsive to mood changes produced by the ambient environment. These authors utilised a randomised cross-over study whereby the plasma levels of AEA, 2-AG, PEA and SEA were measured during the partaking of a meal under conditions of either pleasant or unpleasant ambience. The authors found that levels of SEA and PEA were changed during the experience, and that the lipids were correlated with measures of mood [19]. This raises the question as to whether other acute mood-changing interventions can affect these lipids in a similar manner. Human touch is involved in many rewarding behaviours and is considered to be an inherited, gene specified reward [20]. Touch massage (TM) and human touch reduce stress and anxiety and increase well-being and relaxation in humans [21–24]. In the present randomised cross-over study, we have investigated whether touch massage also affects the plasma levels of eCBs and related lipids.

## Results

### Influence of the interventions upon behavioural and stress-related measures

**Fig. 1:**
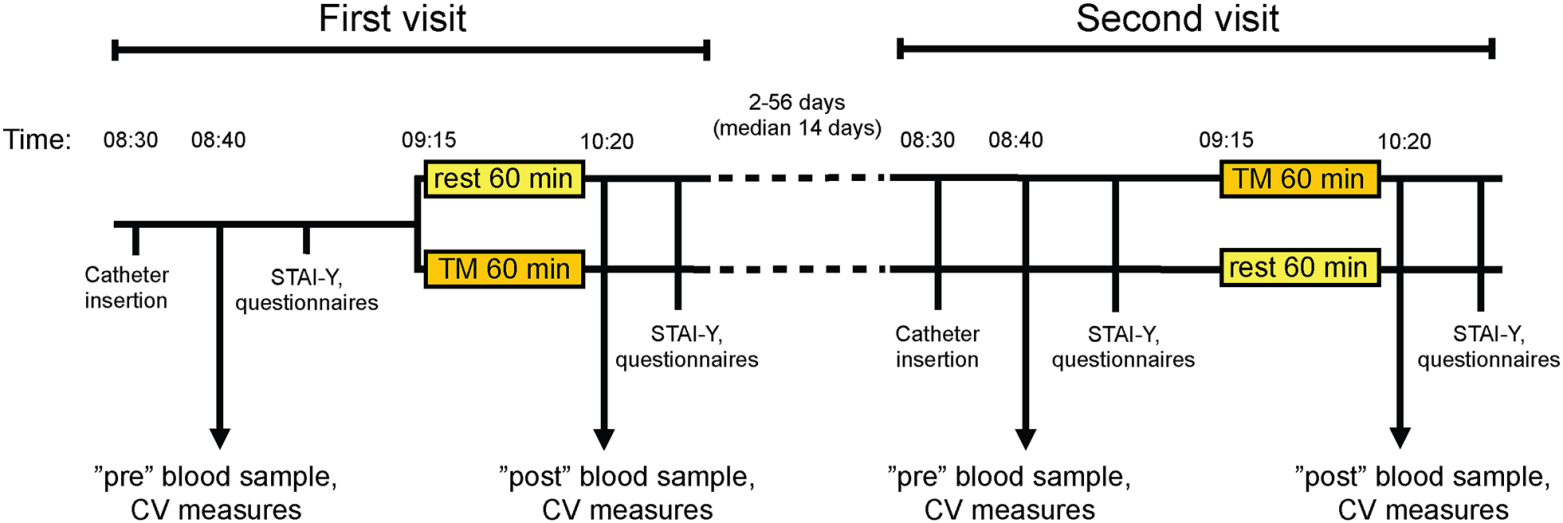
Flow chart of the study showing the sampling times

Twenty healthy individuals participated in the study. The individuals were randomly divided into two groups, one group (N = 10) experiencing a rest phase (60 min) between blood sampling on the first occasion and 60 min of TM between blood sampling on the second occasion. The other group (N = 10) was treated in the reverse order to give a maximum of 20 cases in this randomised cross-over study (for schematic, showing the sampling times, see Fig. 1). The values for all rest and TM conditions were then collated. The initial stress levels (i.e. prior to blood sampling) on a 5 point scale (5 worst) were not significantly different between the rest conditions and the TM conditions, median (range) scores of 1.5 (0–5) and 2 (0–3.5) being found for rest and TM, respectively (P = 0.61, Wilcoxon’s matched pairs signed rank test). Similarly, pre-treatment saliva cortisol levels (nmol) were not different, median (range) scores of 8.8 (4.4–17.2) and 8.7 (3.4–18.6) being found for rest and TM, respectively (P = 0.77, Wilcoxon’s matched pairs signed rank test).

**Fig. 2:**
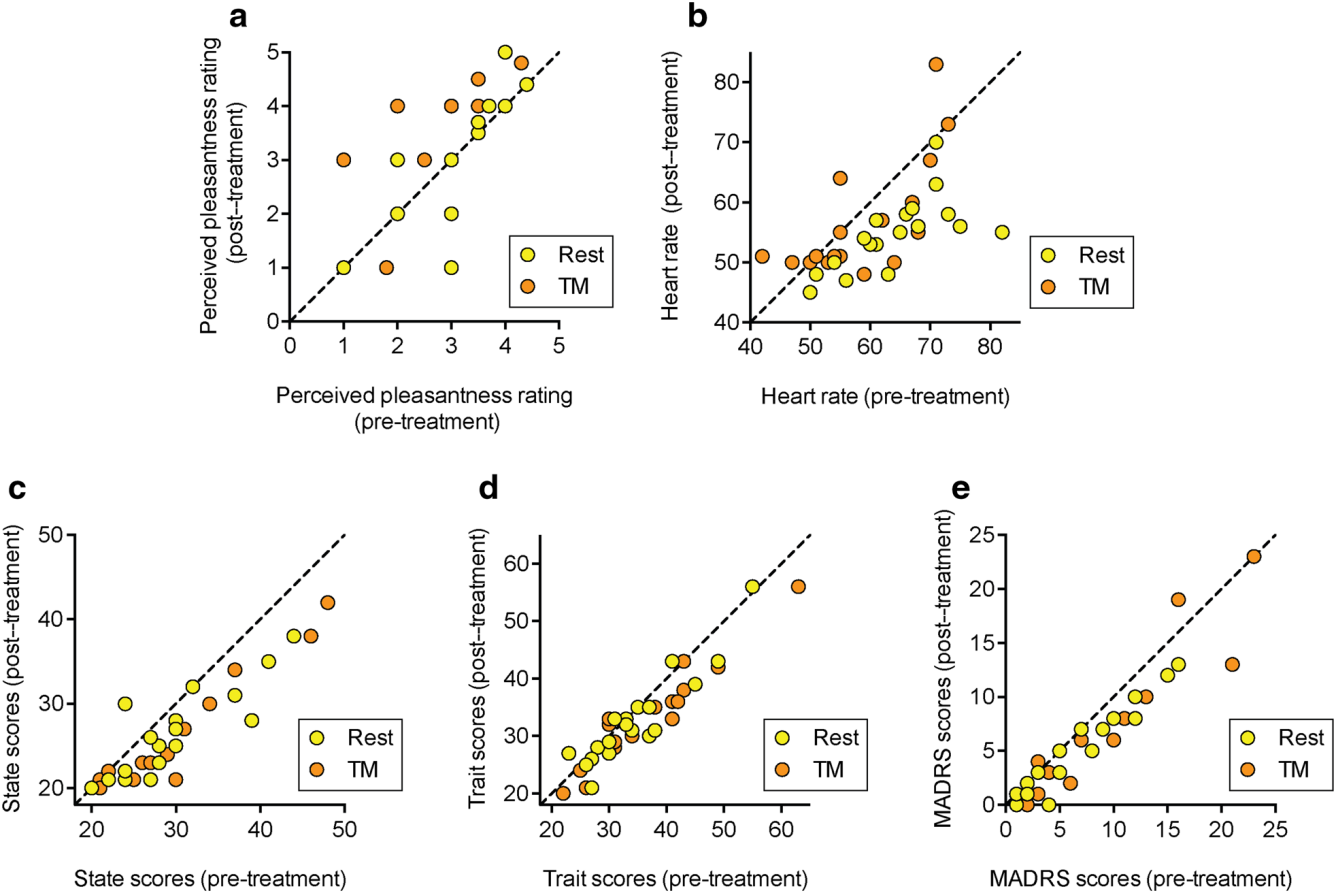
Effects of rest and TM upon **a** perceived pleasantness; **b** heart rate, **c** STAI-state; **d** STAI-trait and **e** MADRS scores of the subjects. The graphs show the individual data points, and the *dotted lines* are the 45° line of identity

The effects of TM upon perceived pleasantness, the heart rate, the total scores in the state and trait arms of the State-Trait Anxiety Inventory Form Y (STAI-Y) and the Montgomery-Åsberg Depression Rating scale (MADRS) are shown in Fig. 2. Each panel shows the individual data points plotted with the x-axis representing the pre-treatment value, and the y-axis representing the post-treatment value. The correlations between the pre- and post-treatment values were very high for all variables (Additional file 1). However, the primary aim of the plots is to provide a simple visual representation of the individual data. In this respect, the dotted 45° lines of identity represent the situation where the intervention had not changed the behavioural scores at all: a treatment effect would result in a consistent deviation away from this 45° line of identity. Significance for treatment effect was assessed using two-way ANOVA for matched samples (Table 1). There was, as expected, a significant treatment × time (pre- vs. post-treatment) interaction for the perceived pleasantness scale (Fig. 2a; Table 1). The median value did not change following rest (median 0, range −2 to +1 units for post- minus pre-treatment values), whereas it increased by a median of 0.5 units (range −1 to +3 units) following TM. A reverse pattern was seen for the heart rate (Fig. 2b): the heart rate dropped by a median of 8 beats/min (range of decrease 1–27 beats/min) following the rest period, whereas the corresponding drop for TM was smaller (3 beats/min, range of decrease −14 to 12 beats/min). Measures of anxiety and depression were also obtained for the participants, with the exception of one case pre-TM, and the behavioural scores are shown graphically in Fig. 2c–e. For the total state scores in the STAI-Y inventory, a significant main effect of time (i.e. pre vs. post-treatment) was seen, but no significant effects of either time × treatment or treatment per se. This can be seen as a downward shift relative to the dotted line in Fig. 2c. Corresponding data for the Trait arm of the STAI-Y scale and the MADRS scale are shown Fig. 2d, e. Taken together, the main effect of TM over and above rest per se in this population was upon perceived pleasantness.

**Table 1:** Statistical evaluation of the datasets shown in Figs. 2 and 3

Measure	N	P value		
		Time	Treatment	Time × treatment
Perceived pleasantness (ranks)	20	0.018	0.018	0.0051
Heart rate (ranks)	19	<0.0001	0.25	0.019
STAI State (ranks)	19	<0.0001	0.31	0.61
STAI Trait (ranks)	19	0.0051	0.78	0.65
MADRS (ranks)	19	0.0019	0.88	0.95
2-AG (log_10_)	18	0.37	0.30	0.67
AEA (sqr)	18	0.36	0.48	0.99
PEA (log_10_)	18	0.64	0.029	0.90
SEA (log_10_)	18	0.13	0.13	0.60
OEA (log_10_)	18	0.39	0.43	0.65
LEA (log_10_)	18	0.59	0.62	0.41
POEA (log_10_)	18	0.67	0.56	0.28
DEA (ranks)	18	0.36	0.74	0.080
EPEA (ranks)	18	0.24	0.27	0.40
NAGly (log_10_)	18	0.86	0.50	0.34

“Time” refers to pre- vs. post-treatment as main effect. Two-way paired ANOVA were conducted either on the ranked data [55] or upon the transformed values using the transformations shown in the Table

*sqr* square root

### Influence of the interventions upon blood levels of eCBs and related lipids

**Fig. 3:**
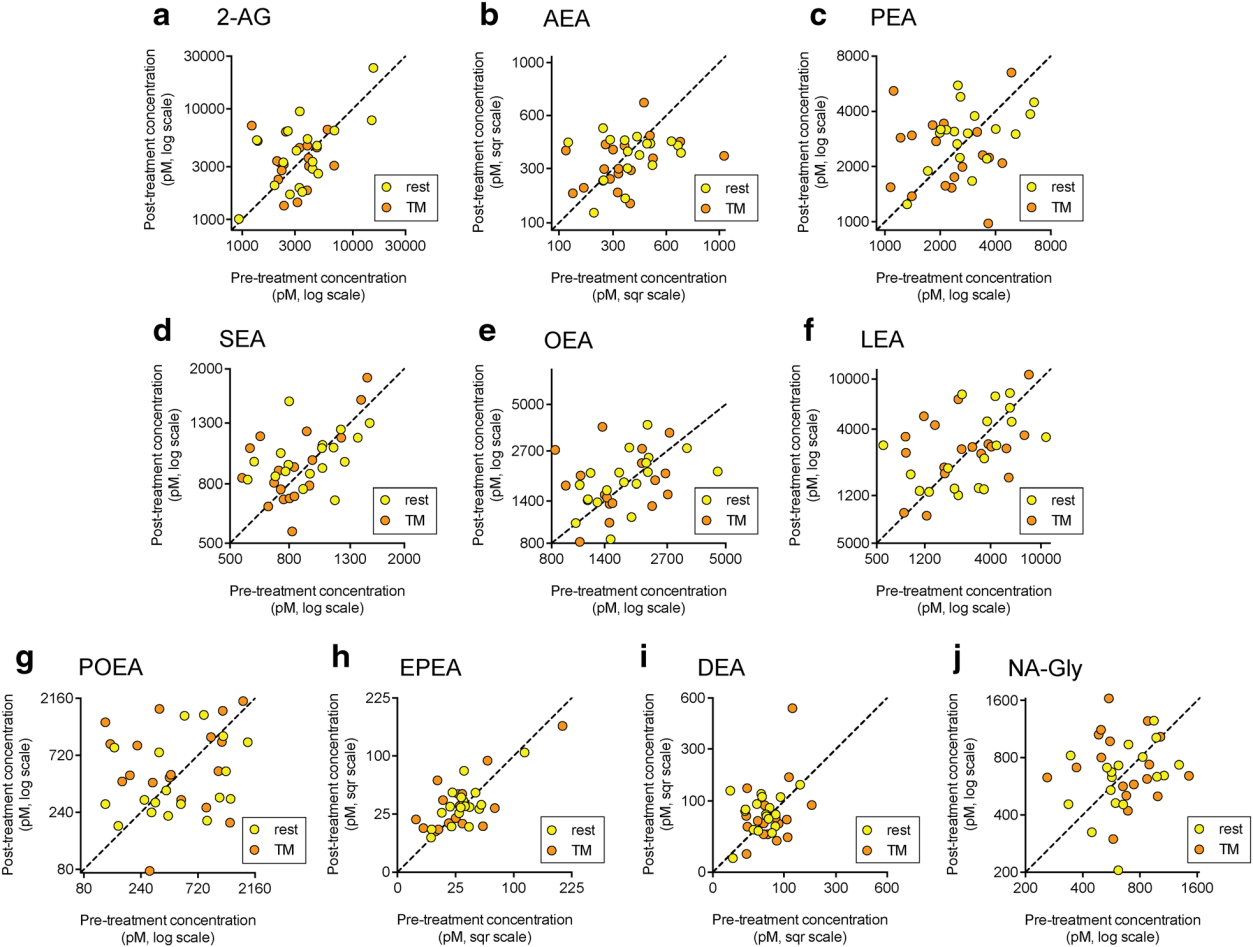
Effects of rest and TM upon the plasma levels of **a** 2-AG; **b** AEA; **c** PEA; **d** SEA; **e** OEA; **f** LEA; **g** POEA; **h** EPEA; **i** DEA and **j** NA-Gly (N = 18). The graphs show the individual data points, and the *dotted lines* are the 45° line of identity

Plasma levels of 2-AG, AEA, the related NAEs PEA, SEA, POEA, OEA and LEA, and the related arachidonoyl derivative NA-Gly are shown in Fig. 3; the statistical treatment of the data are given in Table 1 and the pre-: post-treatment correlation coefficients are given in Additional file 1. One sample was lost for the rest post-treatment group and one for the TM post-treatment group. Thus, eighteen cases were evaluated on all four occasions. For only one compound did all four sample groups pass the D’Agostino and Pearson test for normality (NA-Gly), but this issue was rectified by use of logged values for 2-AG, PEA, SEA, OEA, LEA, POEA and square root values for AEA. Log_10_-transformed NA-Gly also passed the D’Agostino and Pearson test for normality. Two-way ANOVA for repeated measures conducted on the transformed data indicated no effect of time, treatment or the interaction time x treatment other than a significant effect of treatment (P = 0.029) for PEA, due to higher values for the rest occasions than on the TM occasions (Table 1). EPEA and DEA were also measured (Fig. 3h, i). These two compounds failed the D’Agostino and Pearson test for normality both for untransformed data and upon square root transformation. Log_10_ transformation was not undertaken due to the presence of zero (below the limit of detection) values. Two-way ANOVA for repeated measures on the rank-transformed values indicated that there were no effects of time, treatment or time × treatment on the levels of these two lipids (Table 1).

The availability of both biochemical and behavioural data allows analysis of associations to be made using non-parametric Spearman’s correlations. However, use of such a measure assumes that the relative reliability, defined by Baumgartner [25] as the “indication of the degree to which people maintain their position within a group” is acceptable. Here, the definition means the position of the lipid concentration for a given individual within the range seen for the whole sample.

We assessed the relative reliability of the lipids by comparing their pre-treatment values on the first visit (when they were blinded to the forthcoming treatment) and the second visit. The standard methods used to assess relative reliability are intraclass correlation coefficients (ICCs) and Lin’s concordance coefficient (Lin’s CCC), for which the sample sizes here are sufficient [25–28]. These tests are parametric, and so the transformed data were used (Table 2). Fleiss [28] suggested that ICC values <0.4, 0.4–0.75 and >0.75 can be taken to represent poor, fair to good, and excellent reliability, respectively. Using this measure, the data suggest that the measurements for LEA, OEA, POEA and NA-Gly have sufficient relative reliability for investigation of potential associations with the behavioural variables.

**Table 2:** Relative reliability of measurements of endocannabinoids and six related lipids in human plasma samples

Lipid	1st measurement		2nd measurement		P value	Relative reliability (transformed data)	
	Median	IQR	Median	IQR		Lin’s CCC	ICC
2-AG	3245	2024	3300	2541	0.57	0.15 (−0.24 to 0.51)	0.17 (−0.27 to 0.56)
AEA	358	212	364	237	>0.99	0.23 (−0.22 to 0.59)	0.24 (−0.20 to 0.61)
PEA	2572	1595	2074	1565	0.28	0.27 (−0.15 to 0.61)	0.29 (−0.16 to 0.64)
SEA	834	356	789	236	0.37	0.31 (−0.13 to 0.65)	0.33 (−0.11 to 0.67)
OEA	1785	869	1433	1094	0.19	0.46 (0.086 to 0.72)	0.47 (0.06 to 0.75)
LEA	3207	3199	2028	3118	0.17	0.64 (0.32 to 0.83)	0.64 (0.30 to 0.84)
POEA	416	620	342	955	0.45	0.51 (0.12 to 0.77)	0.53 (0.13 to 0.78)
NA-Gly	627	469	617	352	0.84	0.41 (−0.025 to 0.71)	0.43 (0.006 to 0.73)

Median and IQR (interquartile ranges) are for the untransformed data in pM, and the P value comparing the two measurements are from Wilcoxon’s signed ranks matched pairs tests. The Lin’s CCC and ICC (1,1) values are for the transformed data (log_10_ except for AEA where square root was used), required so that the data passed the D’Agostino and Pearson test of normality. Values in brackets are the 95 % confidence limits

In Table 3, Spearman’s rho values for the correlation between the post- and pre-values for LEA, OEA, POEA and NA-Gly, and the corresponding changes for either the state scores or the heart rates, are presented. These were chosen since they are associated with anxiety and stress, and show the largest changes post- : pre-treatment of the variables investigated. None of the zero order correlations reached significance. Table 2 also shows the correlations for “TM-selective” (sTM) change in lipid concentrations vs. the sTM for perceived pleasantness. The sTM values were calculated as the change over time for the TM group minus the change for the rest group, thereby reducing potential influences of time, food intake etc. [19, 29–33]. In this case, significant negative correlations for LEA and POEA were found (Table 3).

**Table 3:** Correlations between plasma lipid, state, heart rate and perceived pleasantness scores

Condition	∆State score	∆Heart rate	sTM perceived pleasantness score		
	Zero order	Zero order	Zero order	First order controlling	
				Gap	BMI
∆OEA					
Rest	0.13	−0.27			
TM	0.41	0.05			
sTM			0.05	0.05	0.03
∆LEA					
Rest	−0.11	−0.26			
TM	0.37	−0.22			
sTM			−0.54*	−0.54*	−0.55*
∆POEA					
Rest	−0.03	−0.44			
TM	0.14	−0.28			
sTM			−0.53*	−0.53^†^	−0.52^†^
∆NA-Gly					
Rest	0.42	−0.10			
TM	−0.01	−0.16			
sTM			0.12	0.12	0.08

The Table shows Zero and first order Spearman’s rho values between the change in plasma levels of LEA, OEA, POEA and NA-Gly and the change in state scores and heart rates after either rest or TM and the TM-selective (sTM) change in perceived pleasantness (N = 18–19). The changes (shown as ∆) were calculated as (post-minus the corresponding pre-treatment value). sTM refers to calculations designed to minimise any effects of time and rest and were calculated as (post-TM minus pre-TM) − (post-rest minus pre-rest). The first order Spearman correlation coefficients controlling for the time elapse between the two measurement occasions (“Gap”) or for the BMI at the start of the study were calculated by the method of Lehmann [56]

* P ≤ 0.025, ^†^ P < 0.05, otherwise not significant

## Discussion

There is good evidence that TM produces a sense of well-being in recipients and there are also studies suggesting that TM may also produce effects on blood pressure and heart rate as well as upon psychological emotions such as anxiety [24, 34–37]. An advantage of the cross-over design used here is that the individuals act as their own controls, and the first author of the present study has previously used this model, with a similar sample size (22 individuals) to demonstrate changes in heart rate and heart rate variability following TM [36]. In the present study, an effect on the perceived pleasantness rating scale for TM was observed. This is consistent with a previous study from the first author, who showed that the increased level of perceived pleasantness was associated with an increased blood flow in the pregenual anterior cingulate cortex [24], a brain region that is activated by pleasurable stimuli [38]. In contrast, effects upon the state arm of the STAI-Y scale over and above that seen for rest was not seen following TM in the present study. The most likely explanation of the lack of effect on this measure is that a floor effect is occurring, given that the volunteers were not overly anxious. Certainly, the TM protocol used here produces a notable reduction in the state scores in patients scheduled for elective aortic surgery [37].

With respect to the biochemical measurements, no significant TM-induced changes, which would be apparent as an interaction time × treatment, were seen at the group level for plasma concentrations of 2-AG, AEA or related lipids following either TM or rest. To our knowledge, the only previous study investigating the response to the eCB system to a hands-on technique is that of McPartland et al. [39], who investigated osteopathic manipulative treatment in non-naïve individuals. They did not find any significant change at the 5 % level in post-treatment serum levels of AEA, 2-AG or OEA compared to pre-treatment levels following either active or sham treatment. The association of the eCB system with stress and anxiety is complex, and a synthesis of animal experimental data has suggested that the activity of this system needs to be kept within a narrow window [40]. In humans, blockade of CB_1_ receptors by rimonabant is associated with an unacceptable incidence of depressive disorders and mood alterations with depressive symptoms and anxiety [18]. Rimonabant given acutely does not affect basal anxiety levels, but increases anxiety under aversive conditions (simulated public speaking) [41]. Conversely, in animal studies, potentiation of the eCB system by compounds inhibiting eCB catabolism produce potentially beneficial effects in models of anxiety using conditions of high, but not low, aversiveness [42–44]. These data may suggest that the eCB system is only engaged once a threshold of anxiety is reached. The type of stress may also be important. Thus, the stress produced by the Trier Social Stress Task (a series of short stressful events including a mock job interview) is sufficient to produce increases in blood levels of AEA, although this may reflect a general increase in NAE levels, since PEA and OEA levels were increased, whereas 2-AG and the related monoacylglycerol 2-oleoylglycerol levels were not changed [15]. Levels of these NAEs were not changed during unstressed conditions [15]. In healthy males participating for the first time in repeat parabolic flights, individuals responding with motion sickness showed an increased stress score, and this was accompanied by a reduction in AEA compared to individuals who did not feel motion sickness after the 10th parabola [45]. These examples are of fairly strong stressors, whereas the individuals in our studywere not unduly stressed or anxious. A lack of effect upon blood eCB levels is thus consistent with the “threshold of anxiety” hypothesis described above.

With respect to the related NAEs, the situation may be different: Schrieks et al. [19] reported that in their randomised cross-over study there were state-dependent changes in PEA and SEA levels in their study investigating the effects of ambience upon plasma levels of these lipids. These authors followed the levels of AEA, 2-AG, PEA, SEA, OEA and docohexanoylethanolamine (DHEA) in plasma at baseline, and 30 and 120 min following the partaking of a meal with or without alcohol and in either a pleasant or an unpleasant atmosphere, created by environmental cues (lighting, cleanliness, choice of music etc.). The authors found a significant interaction effect of time × ambience, so that at the 30 min time point, both PEA and SEA levels (expressed as change from baseline) were lower in the pleasant ambience arms of the study than in the unpleasant ambience arms (both with or without alcohol for SEA, with alcohol only for PEA) whereas the changes in 2-AG and AEA levels were not. It would certainly be worth designing future studies to investigate the effects of TM upon plasma eCB and NAE levels in individuals with higher stress/anxiety levels.

The present study has also investigated whether changes in the concentrations of LEA, OEA, POEA and NA-Gly were associated with changes in the state scores, the heart rates and the “TM-selective” change in perceived pleasantness. At first sight, the results suggest that this may be so, a result in line with the study of Schrieks et al. [19], who found significant correlations with changes between baseline and 120 min of their lipid measurements and mood scores in their questionnaire for the pleasant ambiance without alcohol arm of study. Mood scores in that study were assessed using the Profile of Mood States questionnaire with additional questions from the Brunel mood scale. All in all, seven measures (anger, depression, fatigue, tension, vigour, happiness and calmness) were quantitated and significant positive correlations were found between ∆AEA as well as ∆DHEA and ∆fatigue, and between ∆SEA and ∆calmness, whilst negative correlations were found between ∆2-AG, ∆PEA and ∆happiness, and for ∆OEA and ∆vigour [19]. However, it is wise to remember that the significant correlations from both the present study and that of Schrieks et al. [19] result from multiple testing. In the case of [19] the total number of correlation coefficients that can be obtained from the pleasant ambience without alcohol arm of the study is presumably 42 (6 lipids, seven behavioural scores), and in our case there were 12 zero-order correlation coefficients. There is a trade-off between running the risk of reporting Type I errors (since some of the correlation coefficients would be expected to be significant on a purely random basis) and of reporting Type II errors when conservative corrections, such as the Bonferroni correction, are used. It can be argued that in exploratory studies of this type, the Bonferroni correction is too draconian (for [19] the presumed 42 comparisons for the arm in question would, upon implementation of the Bonferroni correction, reduce the minimum level of significance to 0.0012, which would leave but a single correlation as significant; the same would be true for the less conservative false discovery rate method of Benjamini and Hochberg [46]). Thus, a reasonable conclusion to make is that the significant correlations found in that study and here are consistent with the hypothesis that the circulating levels of endocannabinoids and related NAEs are responsive to environmental cues, but confirmation of these exploratory findings is necessary.

## Conclusions

It is concluded that in the participants studied here, there are no overt effects of TM upon plasma endocannabinoid levels. Possible associations of related *N*-acylethanolamines with the perceived pleasantness should be investigated further.

## Methods

### Ethics statement

This study was approved by the Regional Ethical Review Board in Umeå (Dnr 2014-133-32 M) and the procedures were conducted in accordance with the Declaration of Helsinki. All subjects were informed of the purpose and risks of the experiment before the study and gave their written consent. The study was carried out from September 2014 to February 2015.

### Subjects

Twenty healthy participants [10 men and 10 women, mean age = 25.2 years; standard deviation (SD) = 4.3 and mean body mass index = 23.8; SD = 3.1 at the start of the study] were recruited via posters. Nineteen of the participants were students and one participant worked at the University. Participants were excluded if they were smokers, took medications, or had a history of drug abuse.

### Design

The study had a randomized two-session crossover design with one condition that consisted of TM and a rest condition as control. A sequential number was assigned to an opaque envelope, which contained the assigned treatment group for the participant (half of the participants started with the rest condition and the other half started with the TM condition). The subjects participated twice with a minimum of 2 days and maximum of 56 days apart (median value 14 days; the data was positively skewed). Participants were instructed to have the same breakfast on both occasions and not to take any caffeine in the morning when the study occurred.

### Procedure

The study took place at the same settings and at the same time each day (see Fig. 1 for a flow chart). The participants were prepared with a venous catheter (PVC) at 8.30 a.m. and rested in supine position for 10 min before the blood samples (“pre-measures”) were collected into BD Vacutainer^®^ acrylic based polymer gel tubes containing spray dried lithium heparin. At the same time blood pressure and heart rate were measured. The participants completed questionnaires (see below) before the rest/TM condition, starting at 9.15 a.m. During the “rest” session, the participants rested for 60 min. During the TM session, the participants received TM on legs, back, head, arms and hands for 60 min. The body parts not being massaged were covered with a blanket. The same person that conducted the TM was also present in the room for the rest session.

At 10.20 a.m., blood samples were collected, and blood pressure and heart rate were measured, after which the participants again filled in the questionnaires. All blood samples were centrifuged (15 min at RT) within 10 min of collection, and the plasma was then kept on dry ice until storage at −80 °C. Thus, the pre-treatment samples were placed on dry ice at ~9:10 a.m., the post-treatment samples at ~10:40 a.m. and then delivered to the analysis unit at 11–11:30 a.m. where they were rapidly frozen at −80 °C. Under these conditions, degradation of eCBs has been reported to be minimal, although extended times at room temperature are not recommended [47].

### Intervention

TM is a calm gentle massage with slow (0–5 cm/min) stroking movement at a pressure of about 2.5 N [24, 37]. TM is similar to tactile massage, gentle or soft massage and includes effleurage. There are several different massage techniques, some involving the muscles, others involving the skin. In Sweden, the term TM is used to cover different forms of light massage involving the skin. These forms of massage are characterized by gentle touches of the skin involving light pressure effleurage and long, calm stroking movements intended to increase the well-being [48], pleasure [49] and relaxation [35] of the recipients.

### Subjective measures

To assess subjective effects upon perceived pleasantness and behaviours associated with anxiety, stress and depression, several different instruments were used. Perceived pleasantness was self assessed by the participants by rating how they experienced the moving touch stimulation on a visual analogue scale ranging from −5 (very unpleasant), 0 neutral, and +5 (very pleasant). This scale has previously been used for evaluation of pleasant touch stimulation [24, 37].

The Swedish version of the STAI-Y instrument was used to assess self-reported anxiety levels pre and post intervention/control. The STAY-Y was designed to measure State-Anxiety as a temporary condition of anxiety and Trait-Anxiety as a more underlying condition. The instrument has good construct validity, internal consistency, and test–retest validity [50, 51]. The 20 items are rated on a 4-point scale and after reversing positive items, the total score ranges from 20 to 80, with higher scores reflecting higher levels of anxiety. The STAI-Y instrument is sensitive enough to evaluate anxiety in small groups.

The participants subjective stress levels were rated on a visual analogue scale ranging from 0 = no perceived stress to 5 = very high perceived stress. MADRS was used for subjective experience of depression [52]. The MADRS consists of 9 items rated on a 7-point scale (0–6), which are summated to give a total score.

Heart rate was measured using an automatic blood pressure monitor, M6 Comfort, Omron Healthcare Europe, Netherlands.

### Saliva cortisol

Salivary samples were collected by chewing a cylindrical cotton swab (Salivette, Sarstedt, Numbrecht, Germany). The samples were frozen at −20 °C until assayed. High performance liquid chromatography (HPLC) (Shimadzu Nexera) coupled to liquid chromatography tandem mass spectrometry (LC–MS/MS) (Sciex Qtrap 5500), was used for the analyses. The analyses were conducted in the Clinical Chemical Laboratory at the University Hospital, Umeå, Sweden.

### Chemical and reagents for the lipid analyses

The following native and deuterated standards were purchased from Cayman Chemicals (Ann Arbor, MI, USA): 2-AG, AEA, PEA, SEA, OEA, LEA, POEA, EPEA, DEA, NA-Gly, 2-AG-d_8_, AEA-d_8_, PEA-d_4_, SEA-d_3_, OEA-d_4_, 12-[[(cyclohexylamino)carbonyl]amino]-dodecanoic acid (CUDA). Acetonitrile and methanol were from Merck (Darmstadt, Germany). Isopropanol was from VWR PROLABO (Fontenay-sous-Bois, France). Acetic acid was purchased from Aldrich Chemical Company, Inc. (Milwaukee, WI, USA). Butylhydroxytoluene (BHT) was from Cayman Chemical (Ann Arbor, MI, USA) and ethylenediaminetetraacetic acid (EDTA) from Fluka Analytical, Sigma-Aldrich (Buchs, Switzerland). Glycerol was from Fischer Scientific (Loughborough, UK). All solvents and chemicals were of HPLC grade or higher. Water was purified by a Milli-Q Gradient system (Millipore, Milford, MA, USA).

### Preparation of native lipid standards and deuterated internal standard curves

Analytical quantification standards were used as ready-made standard stock solutions or as solutions prepared from solid substances and stored at −80 °C. 2-AG was prepared and stored in acetonitrile and the other standards were prepared and stored in ethanol. Final stock solution concentrations of native standards were: 250 µg/mL (2-AG, LEA and POEA), 125 µg/mL (AEA, PEA, OEA, DEA, EPEA, NA-Gly) and 83 µg/mL (SEA). Stock solutions of deuterated internal standards were prepared to a final concentration of 40 µg/mL (2-AG-d_8_) and 10 µg/mL (AEA-d_4_, PEA-d_4_, SEA-d_3_, OEA-d_4_).

For each native compound, a suitable internal standard was selected based on structural similarities and added to samples before extraction. The internal standards were: 2-AG-d_8_, AEA-d_4_, SEA-d_3_, OEA-d_4_ (used for POEA, EPEA and NA-Gly as well as OEA) and PEA-d_4_ (used for LEA and DEA as well as PEA). Recovery rates of each internal standard were calculated by normalisation against the recovery standard CUDA added in the last step before injection.

### Sample preparation for lipid analysis

Sample preparation was undertaken according to our previously validated method [53]. Briefly, plasma samples (500 µL) were thawed on ice and spiked with 20 µL internal standard solution (800 ng/mL 2-AG-d_8_, and 20 ng/mL AEA-d_4_, OEA-d_4_, SEA-d_3_ and PEA-d_4_) and 10 μL antioxidant solution [0.2 mg/mL BHT/EDTA in methanol/water (1:1)] and then applied to pre-washed and conditioned solid phase extraction SPE cartridges (Waters Oasis HLB cartridges; 60 mg of sorbent, 30 µm particle size). After loading the sample containing internal standard and antioxidant solution, the cartridges were washed, dried under high vacuum for about 1 min, and eluted with 3 mL acetonitrile, followed by 2 mL of methanol and 1 mL of ethyl acetate into polypropylene tubes containing 6 μL of a glycerol solution (30 % in methanol). Eluates were concentrated with a MiniVac system (Farmingdale, NY, USA), reconstituted in 100 μL of methanol and vortexed (if necessary centrifuged to remove any residuals). Solutions were then transferred to LC vials with low-volume inserts, 10 μL of CUDA (50 ng/mL) was added, and LC–MS/MS analysis was performed immediately.

### Instrumentation used for chromatographic separation and detection of lipids

Lipid analysis was performed using an Agilent UPLC system (Infinity 1290) coupled with an electrospray ionization source (ESI) to an Agilent 6490 Triple Quadrupole system equipped with the iFunnel Technology (Agilent Technologies, Santa Clara, CA, USA) operating in positive mode [54]. Analyte separation was performed using a Waters BEH C18 column (2.1 mm × 150 mm, 1.7 µm particle size) with 10 µL injection volume. The mobile phase consisted of (A) 0.1 % acetic acid in MilliQ water and (B) acetonitrile : isopropanol (90:10). The following gradient was employed: 0.0–2.0 min 30–45 % B, 2.0–2.5 min 45–79 % B, 2.5–11.5 min 79 % B, 11.5–12 min 79–90 % B, 12–14 min 90 % B, 14–14.5 min 90–79 % B, 14.5–15.5 min 79 % B, 15.6–19 min 30 % B.

Precursor ions, [M+H]^+^ product ions, multiple reaction monitoring (MRM) transitions and optimal collision energies were optimized for each analyte. ESI conditions were: capillary and nozzle voltage at 4000 and 1500 V, drying gas temperature 230 °C with a gas flow of 15 L/min, sheath gas temperature 400 °C with a gas flow of 11 L/min. The nebulizer gas flow was 35 psi, and iFunnel high and low pressure RF at 150 and 60 V. The dynamic MRM option was used for all compounds with optimized transitions and collision energies. The MassHunter Workstation software was used to control acquisition and manually integrate all peaks. The intra- and interday precision and accuracy measures of the lipids using this QqQ system are reported in detail elsewhere [54].

### Statistics

Wilcoxon’s signed ranks tests, paired signed ranks tests, non-parametric bivariate correlations, D’Agostino and Pearson normality tests were undertaken using v. 6 for the Macintosh (GraphPad Software Inc., San Diego, CA, USA). Intraclass correlation coefficients and two-way repeated- measures ANOVA were obtained using SPSS software v.22 for the Macintosh (IBM). Lin’s concordance correlation coefficients were calculated online using the facility provided by the National Institute of Water and Atmospheric Research (https://www.niwa.co.nz/node/104318/concordance; URL checked 17 August 2015). The confidence limits for the differences between non-overlapping correlations (Additional file 1) were calculated using a Microsoft Spreadsheet provided by Dr. Thom Baguley at https://seriousstats.wordpress.com/2012/02/05/comparing-correlations/, URL checked 17 August 2015).

## Availability of supporting data

The data sets supporting the results of this article are included within the article.

## Additional files

10.1186/s13104-015-1450-z Correlation coefficients for the pre- vs. post-treatment data summarised in Figs. 2 and 3.

## Abbreviations

2-AG: 2-arachidonglycerol
AEA: anandamide
BHT: butylhydroxytoluene
CB: cannabinoid
CUDA: 12-[[(cyclohexylamino)carbonyl]amino]-dodecanoic acid
DEA: docosatetraenoyl ethanolamide
eCB: endocannabinoids
EPEA: eicosapentaenoyl ethanolamide
ESI: electrospray ionization source
HPLC: high performance liquid chromatography
ICC: intraclass correlation coefficient
LC-MS/MS: liquid chromatography tandem mass spectrometry
LEA: linoleoyl ethanolamide
Lin’s CCC: Lin’s concordance coefficient
MRM: multiple reaction monitoring
NA-Gly: *N*-arachidonoylglycine
NAE: *N*-acylethanolamine
OEA: oleoylethanolamide
PEA: palmitoylethanolamide
SEA: stearoylethanolamide
sqr: square root transformation
STAI-Y: State-trait anxiety inventory form Y
sTM: TM-selective
TM: touch massage
