# Supplementary material for: Endocannabinoids and related lipids in blood plasma following touch massage: a randomised, crossover study
Source: BMC Res Notes. 2015 Sep 29;8:504. doi: 10.1186/s13104-015-1450-z (PMC4589181; doi:10.1186/s13104-015-1450-z)
Supplement: Supplementary file 1 — 10.1186/s13104-015-1450-z Correlation coefficients for the pre- vs. post-treatment data summarised in Figs. 2 and 3. [file 13104_2015_1450_MOESM1_ESM.docx]

**Additional File 1. Correlation coefficients for the pre*- vs.* post-treatment data summarised in Figs. 2 and 3.**

|  |  |  |  |  |  | |
| --- | --- | --- | --- | --- | --- | --- |
|  | Pearsons/^†^Spearman's r | | Lin's CCC | | ICC |  |
| Treatment | Rest | TM | Rest | TM | Rest | TM |
|  |  |  |  |  |  |  |
|  |  |  |  |  |  |  |
| Perceived pleasantness (ranks) | 0.90^†^ (0.75 to 0.96)*** | 0.81^†^ (0.56 to 0.92)*** |  |  |  |  |
| Heart rate (ranks) | 0.74^†^ (0.43 to 0.90)*** | 0.68^†^ (0.32 to 0.86)** |  |  |  |  |
| STAI state (ranks) | 0.87^†^ (0.68 to 0.95)*** | 0.92^†^ (0.80 to 0.97)*** |  |  |  |  |
| STAI trait (ranks) | 0.87^†^ (0.69 to 0.95)*** | 0.94^†^ (0.85to 0.98)*** |  |  |  |  |
| MADRS (ranks) | 0.94^†^ (0.85 to 0.98)*** | 0.95^†^ (0.88 to 0.98)*** |  |  |  |  |
|  |  |  |  |  |  |  |
| 2-AG (log_10_) | 0.62 (0.24 to 0.84)** | -0.065 (-0.54 to 0.40)^NS^ | 0.61 (0.24 to 0.83) | -0.061 (-0.47 to 0.37) | 0.63 (0.27 to 0.84) | -0.061 (-0.48 to 0.39) |
| AEA (sqr) | 0.51 (0.066 to 0.78)* | 0.19 (-0.29 to 0.59)^NS^ | 0.48 (0.077 to 0.75) | 0.17 (-0.25 to 0.54) | 0.50 (0.083 to 0.77) | 0.18 (-0.28 to 0.57) |
| PEA (log_10_) | 0.36 (-0.11 to 0.70)^NS^ | -0.050 (-0.49 to 0.41)^NS^ | 0.35 (-0.098 to 0.68) | -0.049 (-0.47 to 0.39) | 0.37 (-0.82 to 0.70) | -0.034 (-0.46 to 0.41) |
| SEA(log_10_) | 0.38 (-0.094 to 0.71)^NS^ | 0.58 (0.17 to 0.81)** | 0.36 (-0.074 to 0.68) | 0.53 (0.15 to 0.77) | 0.38 (-0.053 to 0.71) | 0.53 (-0.072 to 0.70) |
| OEA (log_10_) | 0.55 (0.13 to 0.80)* | 0.33 (-0.14 to 0.68)^NS^ | 0.55 (0.14 to 0.80) | 0.33 (-0.13 to 0.67) | 0.56 (0.15 to 0.81) | 0.35 (-0.11 to 0.68) |
| LEA (log_10_) | 0.59 (0.18 to 0.82)** | 0.28 (-0.20 to 0.65)^NS^ | 0.58 (0.20 to 0.81) | 0.28 (-0.19 to 0.64) | 0.60 (0.22 to 0.82) | 0.30 (-0.16 to 0.65) |
| POEA (log_10_) | 0.34 (-0.13 to 0.69)N^S^ | 0.090 (-0.38 to 0.52)^NS^ | 0.33 (-0.12 to 0.67) | 0.084 (-0.34 to 0.48) | 0.36 (-0.096 to 0.69) | 0.079 (-0.37 to 0.50) |
| DEA (ranks) | 0.060^†^ (-0.42 to 0.51)^NS^ | 0.32^†^(-0.17 to 0.69)^NS^ |  |  |  |  |
| EPEA (ranks) | 0.47^†^ (0.0034 to 0.77)* | 0.43^†^ (-0.047 to 0.75)^NS^ |  |  |  |  |
| NAGly (log_10_) | 0.48 (0.030 to 0.77)* | -0.11 (-0.53 to 0.37)^NS^ | 0.47 (0.043 to 0.75) | -0.10 (-0.50 to 0.34) | 0.49 (0.069 to 0.77) | -0.10 (-0.52 to 0.35) |
|  |  |  |  |  |  |  |

Data are for the transformations shown (sqr, square root). Numbers in brackets are the 95% confidence interval. For the Pearson’s and Spearman’s rho values, ***P<0.001, **P<0.01, *P<0.05, ^NS^P>0.05. It is of course important to demonstrate the significance of the difference between the values for rest and TM, rather than the difference in the significances [57]. In the case of the Pearson’s r values, there are methods available to do this for correlated but non-overlapping dependent correlations (nomenclature of [58]). Using the method of [58], the 95% confidence interval for the difference for Pearson’s r values between pre-rest-post and pre-TM-post were: 2-AG, 0.38 to 0.97; AEA, 0.01 to 0.61; PEA, 0.06 to 0.73; LEA, 0.02 to 0.60; SEA, -0.48 to 0.07; OEA, -0.06 to 0.50; POEA, -0.16 to 0.32; and NA-Gly, 0.26 to 0.87.
